# Perceptions and Attitudes of Argentine Zoomers towards Sustainable Food Production

**DOI:** 10.3390/foods12051019

**Published:** 2023-02-27

**Authors:** Andrea Beatriz Damico, Yari Vecchio, Margherita Masi, Jorgelina Di Pasquale

**Affiliations:** 1 Department of Veterinary Medicine, University of Teramo, 64100 Teramo, Italy; 2 Faculty of Agricultural Science, National University of Lomas de Zamora, Buenos Aires 1836, Argentina; 3 Department of Veterinary Medical Science, University of Bologna—Alma Mater Studiorum, 40064 Bologna, Italy

**Keywords:** Zoomers’ behaviour, sustainable food production, knowledge of sustainability, climate change, consumption of sustainable foods

## Abstract

Young people are concerned about climate change. Their activism has attracted the attention of the media and politicians. Some of them are entering the market as consumers for the first time and can express their preferences without parental guidance: they are the Zoomers. Do these new consumers have enough knowledge about sustainability to be able to make choices in line with their concerns? Are they able to push the market towards change? A sample of 537 young Zoomer consumers were interviewed face-to-face in the Metropolitan Area of Buenos Aires. They were asked to indicate their level of concern for the planet and the first word they associated with sustainability, to rank in order of importance sustainability-related concepts and to indicate their willingness to buy sustainable products. The results of this study underline a high concern for the health of the planet (87.9%) and for unsustainable production methods (88.8%). However, the respondents perceived sustainability as consisting of a single main pillar, the environmental dimension (with 47% of the terms referring to sustainability), and two accessory pillars, the social (10.7%) and economic (5.2%) dimensions of sustainability. Respondents also showed a high interest in products obtained through sustainable agriculture, with a high percentage of them willing to pay for these products (74.1%). However, a substantial correlation was found between the ability to comprehend the notion of sustainability and the determination to purchase sustainable items, and vice versa, between those who reported difficulty comprehending the concept and their determination not to purchase these products. Zoomers believe that the market must support sustainable agriculture through consumer choices without paying a premium price. Clarifying the concept of sustainability, enhancing knowledge and assisting consumers in identifying sustainable products, as well as marketing them at reasonable prices, are essential actions for fostering a more ethical agricultural system.

## 1. Introduction

Do young consumers have enough knowledge about sustainability to promote sustainable production through their food choices? Sustainability is a broad, complex and multidisciplinary topic [1,2] based on three main dimensions, the “environmental”, “social” and “economic” pillars, as reported widely in the literature [3,4,5,6]. Consumers’ knowledge about the concept of sustainability may impact their purchasing decisions, yet some may be put off by the difficulty of confronting such a vast and intricate topic.

Greater awareness about sustainability makes consumers worry about how food is produced [7] and become increasingly demanding, especially in terms of, socio-ethical and environmental issues [8,9].

In recent years, youth movements in favour of the planet, in particular, the Fridays for Future movement [10] and the Rise for Climate demonstration [11], have emerged. More and more young people, in comparison to older generations, express anxiety about the future as a result of the effects of climate change. Some of the young people who identify with these movements are still dependent on their parents for financial support, as they are still in school and do not yet have their own households. The young people who face the market for the first time belong to generation Z and today are between 18 and 27 years old. As Priporas et al. [12] (p. 375) highlight, “this generation seems to be the biggest future marketing challenge”. This generation includes people who begin to buy food based on their preferences, as opposed to school students, who are primarily influenced by their parents’ choices [13]. Because its members are frequently exposed to smart devices (such as mobile phones and tablets) in multiple areas of their lives from a young age, this generation is also known as the digital native generation. As pointed out by Özkan and Solmaz [14] (p. 222), “this situation has also changed the perception of time and space in consumption habits” and determined a particular condition, which is different from the preceding generations.

Numerous studies focus on the behaviour of a cohort or generation [2,15,16,17,18]. Initially, the cohort was a demographic notion that had both social and personal referents [19], that is, people who followed a similar life path, such as those born in the same year, with a duration of 20 to 25 years, or the time it generally takes for a group to be born, grow up and have children of their own, or enter a particular system in a given year [20,21,22]. The idea of a literal birth cohort has been progressively replaced with the concept of a cohort of people united by having experienced (or been exposed to) common events of a given historical period or social events, such as a war, the nuclear bomb, the Chernobyl disaster or a pandemic. These experiences and events instil individuals within each group with similar values, attitudes and beliefs that distinguish them from other cohorts [22,23]. “Thus, cohorts are people about the same age who in a given period have similar experiences that may affect them the same way” [24] (pp. 66–67).

The present research examined the perception and knowledge of sustainability of young consumers belonging to generation Z (Zoomers) and living in the Metropolitan Area of Buenos Aires (AMBA, for its initials in Spanish), Argentina, in order to understand how to promote sustainable food production.

It is important to know whether Argentine consumers have enough knowledge about sustainability and/or concern for the planet to promote food choices of “sustainable production”. While much research (e.g., Ref. [7]) has used multivariate analysis models to examine how specific sociodemographic characteristics influence consumer behaviour, this study takes a different perspective. In other words, we aim to determine whether the purchasing choices of new consumers favour production processes that incorporate “sustainability” as a differentiating quality and an added value, generating interest in both the agri-food sector and the development of public policies that improve the lives of citizens in present and future generations. The development of a sustainable primary sector and a sustainable food transformation process, beyond the national market, is particularly relevant as Argentina is one of the world’s leading producers of raw materials and foods of agricultural and livestock origin.

The present work contributes to filling a knowledge gap, since no other studies analyse, in terms of sustainability, the behaviour of young Argentine consumers belonging to generation Z.

The present work is structured as follows:

Section 1. Introduction, Section 1.1 theoretical background on consumer perception and purchasing behaviour;

Section 2. Materials and Methods, Section 2.1 Materials, Section 2.2 Methods;

Section 3. Results; Section 3.1. Demographic characteristics, Section 3.2. Concern for sustainability, Section 3.3. Perception of sustainability; Section 3.4. Perception of sustainable production and food;

Section 4. Discussion;

Section 5. Conclusions.

### 1.1. Theoretical Background on Consumer Perception and Purchasing Behavior

Consumer perceptions and choices have a great impact on what and how food is produced [25,26]. The awareness that the choices, behaviours and lifestyles of consumers, or their consumption decisions, play a key role in achieving sustainable development is one of the greatest agreements that has emerged over the last decade [27,28,29,30,31]. The concept of “sustainable production” is somewhat nebulous, since sustainability can be understood as the need to decrease the environmental impact by reducing productivity as occurs in organic agriculture, or by increasing productivity through production intensification and improvement as in the case of Life Cycle Assessments. However, many consumers see it as a positive trend [25]. In general, consumers’ positive attitudes towards sustainable and ethical products lead to perceptions of better taste, quality and safety, and of health, environmental and regional economic benefits [8,32]. These attributes can both stimulate consumption and favour sustainable production. Some consumers, interested in the effects of their food choices on the environment, adopt more eco-friendly eating habits [2]. This generates positive impacts on the environment (such as the supply of organic products with a lower carbon footprint or with fewer food miles, etc.), or on the socio-ethical component (such as Fair-Trade products, with greater respect for animal welfare, etc.), the latter gaining relevance in purchasing decisions [2,8,33,34].

Unfortunately, behaviour patterns are not always unambiguously consistent with attitudes [8], and therefore there could be a gap between what is “right” and what is “liked” or, in some cases, within the purchasing capacity. Foods produced sustainably tend to have higher costs than their counterparts from traditional production. This is why, despite their positive attitudes, middle- and low-income consumers may be unable to buy sustainable food products and the diffusion of such products may be hampered [2,35].

A recent review has shown that budget constraint seems to be one of the main factors limiting the choice to purchase green products, and that the attitude towards purchasing these products decreases with the increase in the number of children. However, there is still a greater propensity to purchase green products by women than men [36]. Obviously, since they are dealing with data from questionnaires and not real purchasing behaviour, it should be remembered that the literature has amply demonstrated the overestimation risk, a psychological phenomenon that falls under the social desirability bias, that implies a significant difference between declared behaviour and real behaviour [37,38].

It must also be considered that, changing consumers’ eating habits entails a lengthy and difficult process, creating a further challenge [39].

It is therefore necessary that the primary sector and the agri-food industry must propose new paradigms in the supply of products, providing solutions and alternatives to new consumers, especially youngsters [40] who are looking for answers to their purchasing habits that are consistent with their ethical convictions. Although sustainable alternatives can be imposed through regulations or laws, it is important that market forces act, either through the choices of consumers themselves, who prefer more sustainable alternatives [25], or through the actions of companies, since companies must address the issue of sustainability. According to Kotler [41] (p. 132) “Companies need to make drastic changes in their research-and-development, production, financial, and marketing practices if sustainability is to be achieved”.

## 2. Materials and Methods

### 2.1. Materials

This work was developed on the basis of three research hypotheses:

**H1.**  *The concept of sustainability is not yet widely understood.*

**H2.**  *There is an environmental sensitivity that is reflected in the more “eco-friendly” purchasing decisions of the new generations.*

**H3.**  *That the Zoomers, being the latest generation that has entered the market independently, can represent a challenge for companies that have decided to change their production paradigm by offering new, more sustainable solutions.*

In order to answer these questions, between May and July 2022, a survey was conducted among young consumers belonging to the Zoomer generation, aged between 18 and 27 (born between 1995–2004). The interview was conducted face-to-face, with a probabilistic, judgmental sampling that included only Zoomer consumers. The interviews were conducted on different weekdays and at different times of day, near markets and supermarkets and at bus and train stations, in different geographic locations within AMBA. Participation in the survey was voluntary, subject to informed consent and the declaration of being of age. The questionnaire had closed-ended questions with categorical answers and metric scales (from 0 to 10). A pre-test and a pilot test were carried out among a small group of people (*n* = 10) belonging to the target population, to correct any errors, inconsistencies and repetitions.

The survey consisted of 43 questions grouped into four thematic sections:

1. Demographic characteristics: This section comprised questions about demographic information and the type of dietary choice.
2. Concern for sustainability: This section inquired into the degree of concern for the sustainability of the planet and the long-term use of resources, as well as the source of information that the participants used most often.
3. Perception of sustainability: This section included questions about the association between the idea of sustainability and a list of proposed terms; the concept of sustainability; the respondents were asked to rank in order of importance 12 statements representing the three dimensions of sustainability most referenced in the literature (environmental, social and economic).
4. Perception of sustainable food production: This section concerned the respondents’ perceptions about the degree of sustainability in food production in Argentina, both in general (at the national level) and in the different production chains; the characteristics of sustainable products; the different factors that could favour the development of sustainable food; and the respondents’ purchase intention to buy sustainable food.

The database was managed with Microsoft Excel (Microsoft Corporation, Albuquerque, NM, USA), and the Infostat Version 2020 (Microsoft Corporation, Albuquerque, NM, USA) and SPSS v. 28 (IBM, Armonk, NY, USA) software programmes were used for the quantitative analysis [42].

### 2.2. Method

The first stage of the study involved a descriptive analysis using frequency tables, in order to characterise the sample demographically and provide general information about the AMBA Zoomers. The second stage consisted of bivariate quantitative analyses to find relationships between pairs of variables and to determine the statistical significance of the possible differences observed [43]. As the aim of the research is to understand the relationship between the determination to buy sustainable products and the ability to understand the multidimensional concept of sustainability, the Analysis of Variance (ANOVA) is the statistical method chosen to explain this phenomenon. Indeed, ANOVA represents a useful method to test a linear relationship between groups. Table 1 summarises the set of statements evaluated through a Likert-type scale with a minimum score of 0, meaning “I do not agree”, and a maximum score of 10, meaning “I strongly agree”.

The statements were divided into three categories: social, environmental and economic values. These statements were worded using affirmative and negative forms to check the reliability of the data and not to risk a bias towards either end of the scale. Answers were framed within the same response modes and calculated with a uniform Likert-type scale, grouped through an optimisation process. For this reason, a transformation was carried out by inverting the answers of the negative statements, so as to have uniformity of judgement for all the answers. For example, the statement “Sustainability also gives rise to a profitable activity that creates work” was considered in its opposite meaning, as “Sustainability does not give rise to a profitable activity that generates work”. Then, in order to calculate the value of each of the categories, we proceeded with the following formula:

(1) $$y_{c}=\frac{\sum_{i=1}^{n}x_{ic}}{\sum_{i=1}^{n}max\;(x_{ic})}$$

where *y_c_* is the category to be calculated; *x_ic_* is the observed values of the statements belonging to the category; and max *x_ic_* is the maximum value of the sum of the values of the applications belonging to the category. The values of y ranged between 0 (when all values were 0 on the scale) and 1 (when all values of the variable were equal to 10 on the scale). The process was repeated for all three categories. After verifying the absence of correlation and the internal consistency between variables, the three indicators—social values, environmental values and economic values—were calculated according to the equation above. The use of the indicators allowed us to estimate a synthetic value, where each variable equally contributes to the formation of the values. In order to estimate the propensity to buy eco-friendly products, a one-way ANOVA was performed. Therefore, two groups of variables were included in the ANOVA test:

- Grouping factor: Consumers who are highly determined to buy eco-friendly products, and consumers who are extremely determined not to buy eco-friendly products
- Dependent variables: social, environmental, and economic values

The ANOVA is a statistical model used to test the differences in a continuous variable among two or more groups in a sample. In particular, the null hypothesis H0 states that the average value is the same for all the groups; the alternative hypothesis H1 is that the average value is not the same for all the groups. The statistic used in the ANOVA is the F-test, computed through the ratio of between-group variance to within-group variance. If the null hypothesis is true, the value of the ratio is 1; otherwise, if the alternative hypothesis of different group means holds true, the F value will be greater than 1. The critical values of the F-statistic for which we can reject the null hypothesis are established in a table of probability values for the F-distribution, depending on the level of significance and the degrees of freedom. If the computed F-statistic is smaller than the one reported in the table, then we accept the null hypothesis, while if the F-statistic is greater than the critical value, we have statistically significant evidence that there is a difference in group means. To validate the quality of the analysis, robust tests on the mean were performed. Based on the characteristics of the variables, we used the Brown-Forsythe and Welch tests. The significance was set at a *p*-value of 0.05 for all the tests.

## 3. Results

### 3.1. Demographic Characteristics

The sample consisted of 537 young Zoomer consumers (aged between 18 and 27; 67.2% female), from the Metropolitan Area of Buenos Aires City. They were inquired about their level of education, occupation, household, and type of diet; results are summarized in Table 2.

### 3.2. Concern for Sustainability

After the demographic characterisation, the respondents were asked about their concern for the planet and subsequently, interspaced with other questions, about their concern for the use of resources: 87.9% of Zoomers expressed high concern for the planet, 11.0% moderate concern and only 1.1% low concern (Table 3). In addition, 88.8% of the respondents reported being highly concerned about food production methods, 9.5% moderate concern and 1.7% low concern (Table 3). It is important to note that, although the two statements were not presented together but were spaced out, the answers overlapped on the level of concern expressed.

**Figure 1:**
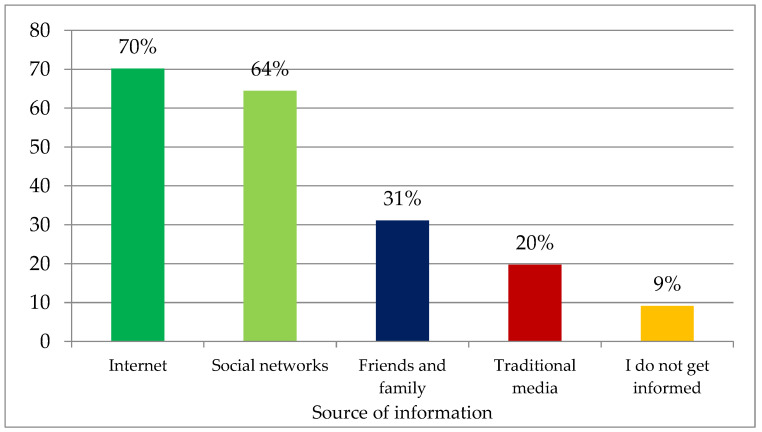
Sources of information used by interviewees (%).

Concerning the sources of information, 70.2% of the interviewees reported that they obtained information on the Internet and through social networks, and only 19.7% used traditional means of communication (television, newspapers, magazines, books) to obtain information on the subject (Figure 1).

### 3.3. Perception of Sustainability

The interviewees were asked to indicate the three words most closely related to the term “sustainability” from a list of 12 options (Table 4). It turned out that the words associated with the environmental dimension were the options selected by most respondents (47%), followed by crosscutting words (37.1%).

After identifying the words most related to sustainability, the respondents were asked to indicate how easy it was to understand the concept of sustainability. Only 32.8% of those interviewed indicated that it was an easy concept to understand, while the remaining 67.2% believed that it was moderately difficult or very difficult to understand (Table 5).

Successively, 12 statements related to sustainability were proposed (without informing to which dimension each phrase belonged), and the respondents were asked to indicate the degree of importance they attributed to each statement. All statements obtained high ratings, with average values above 7 (Table 6).

In order to obtain a generic assessment of each of the three dimensions of sustainability most widely quoted in the literature (social, environmental and economic), the mean values were calculated. Regarding the degree of importance, the dimension most highly valued was the environmental one with an average of 9.1, followed by the social dimension with an average of 8.4 and, lastly, the economic dimension with an average of 7.9 (Table 6).

### 3.4. Perception of Sustainable Production and Food

**Figure 2:**
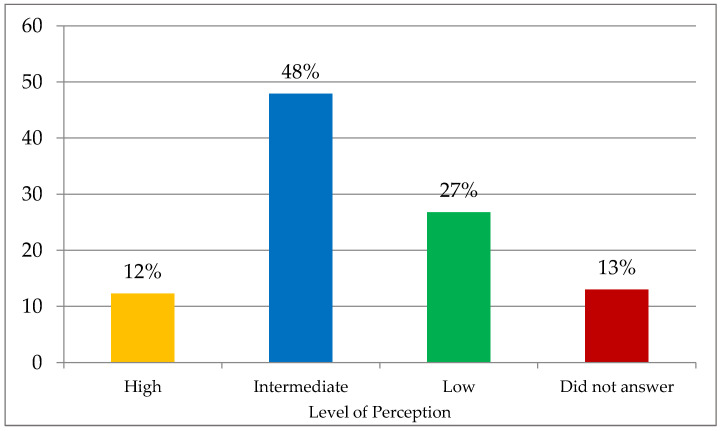
Perception of food production sustainability in Argentina (%).

Regarding the perceived level of sustainability of the Argentine production system (Figure 2), the results indicate that slightly under half of the interviewees (47.9%) perceived the country’s production as having an intermediate level of sustainability, while about 13% did not answer.

**Figure 3:**
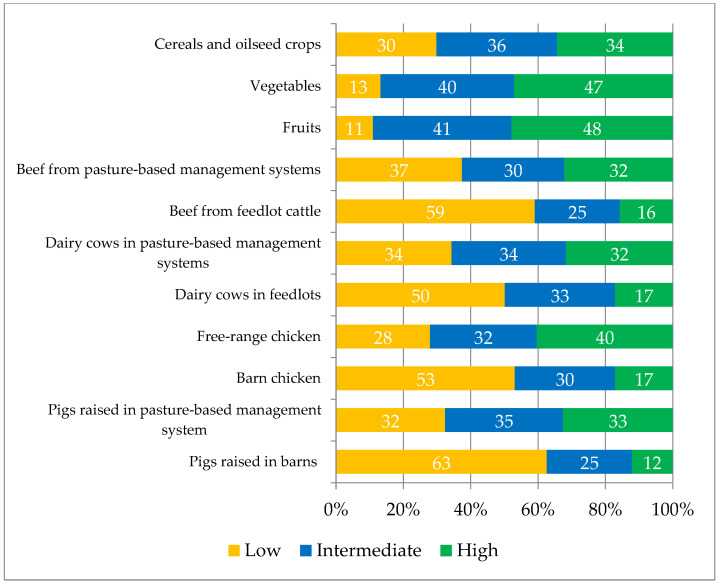
Assessment of the degree of perceived sustainability of different production chains.

Subsequently, the respondents were asked to assess the different food production chains, based on their perception (Figure 3).

In general, the production of vegetables was perceived as more sustainable (average ratings between 5.2 and 6.2) than that of foods of animal origin (average between 2.9 and 5.4). In addition, vegetable production performed on large amounts of land was perceived as less sustainable.

In particular, among vegetable production systems, horticulture (intensive production) and fruit growing (semi-intensive/semi-extensive production) were assessed as highly sustainable by almost half of the respondents (47.1 and 47.9, respectively). Grain production (cereals and oilseeds) was associated with an intermediate level of sustainability by 35.9% of the respondents.

Regarding production of animal origin, all of the proposals listed were perceived to have a low level of sustainability, except for chicken, pigs and cows raised in pasture-based management systems, where sustainability was rated as high and intermediate by most respondents (40.4%, 32.6%, and 32.2% respectively).

**Figure 4:**
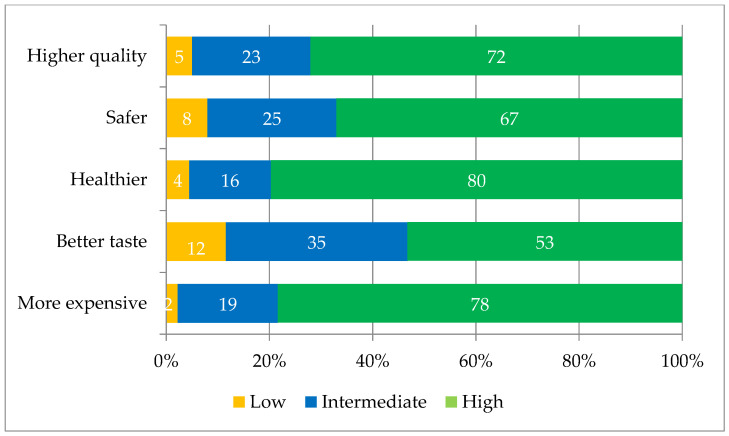
Assessment of the perceived attributes of sustainable food (%).

Regarding foods derived from sustainable production systems, respondents were asked about the attributes that differentiate them from conventional products. Sustainable foods were perceived as having superior characteristics compared to conventional products (Figure 4). These foods were identified as “healthier” (79.7%) with a mean score of 8.0; of “higher quality” (72.1%) with a mean score of 7.6; “safer” (67%) with a mean score of 7.3; having “better taste” (53.3%) with an average score of 6.6; and “more expensive” (78.4%) with an average score of 8.0 (Figure 4).

Different factors may favour sustainable production of food, such as the choices of consumers and the policies of the sector. When asked about these factors, the respondents indicated that it was desirable for the market to act, that consumers can promote changes in the production system and favour more sustainable products through purchase choices (59.2%), that their choices should be fostered by the presence of certification (54.2%) or could be driven by state policies (48.2%) and, to a lesser extent, that consumers should pay a higher price for this type of food (46.0%) (Table 7).

**Figure 5:**
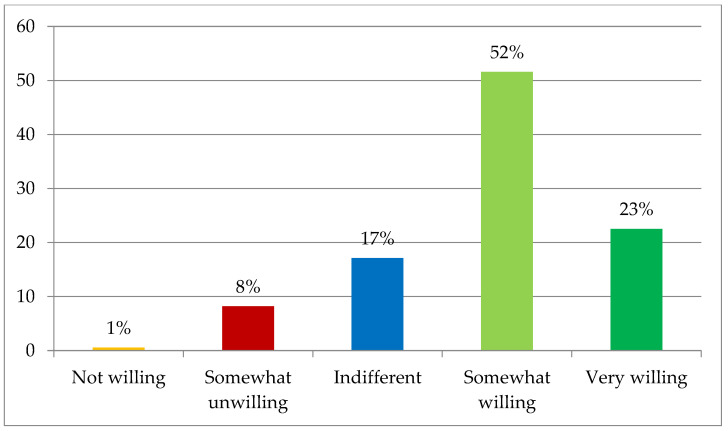
Willingness to purchase sustainable products (%).

Although less than half of the sample (46%) indicated that consumers should agree to pay a premium price for sustainable products in order to incentivise the market and sustainable production (Table 7), more than 74.1% stated that they would personally be willing to pay a higher price (Figure 5). However, it should be noted that only 22.5% seemed to be actually determined to buy more sustainable products, despite having to pay more for them, while 25.9% did not indicate purchase intention. Most of the interviewees (51.6%) showed a half-hearted willingness to pay a higher price for sustainable products.

Pairwise comparisons between consumers who were very willing to buy sustainable products (“Determined to buy”) and those who were not willing (“Determined not to buy”) show that the respondents that wish to consume sustainable products have a statistically higher perception of the environmental, social, and economic impacts of these products (Table S1, see Supplementary Materials).

The existence of statistically significant differences between the different groups considered is demonstrated by a highly significant *p*-value (less than 0.05). Therefore, the two groups can be considered different with regard to their perception about this dimension. The two robustness tests of Welch and Brown–Forsythe carried out confirmed the possibility of accepting the null hypothesis since the significance of the test was less than 0.05 (Table S2, see Supplementary Materials).

Young consumers’ perception of specific aspects of these products certainly contributes to determining their degree of determination to buy sustainable products, with significant differences between the groups (<0.05) (Table 8). The results show that those determined to buy sustainable food products perceived them to be of better quality and healthier. This finding brings to light the behaviour of the believers in sustainable food, who cement their determination not only in the environmental or social attributes that these products have, that is, the external context, but also in the attributes that directly impact them, finding these products to be of higher quality than others.

**Figure 6:**
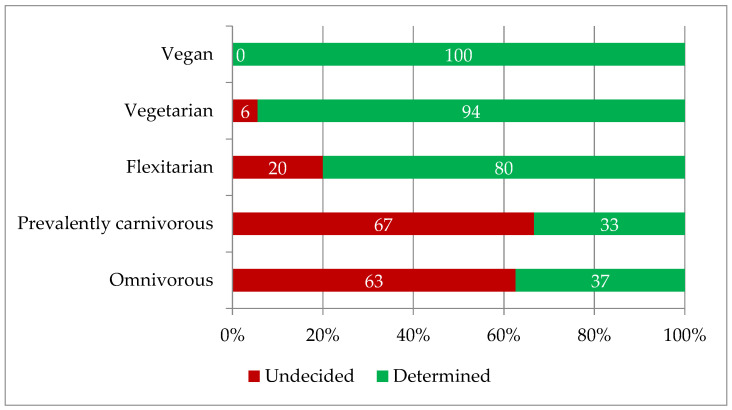
Distribution of the interviewees in two groups, “Undecided” and “Determined”, based on their food choices.

Furthermore, analysis of the food diet choices reveal that individuals who have adopted more animal-friendly diets, such as flexitarians, vegetarians and vegans, are also those more strongly determined to buy such products, while those who consume more traditional food diets showed strong indecision (Figure 6).

Finally, a statistically significant relationship was found between the determination to buy sustainable products and the ability to understand the concept of sustainability. The consumers who expressed greater determination to buy sustainable products indicated that the concept of sustainability was clear and easy to understand. In contrast, those undecideds to buy sustainable products believed that the concept of sustainability was not easy to understand (Table S3, see Supplementary Materials).

## 4. Discussion

Slightly under 90% of the young people interviewed were strongly concerned about the production methods currently used and the consequent sustainability of the planet. This result is in line both with the high level of awareness of young people regarding the severity of the planetary crisis and with the high concern for their own future and that of future generations [45,46,47] that has strongly emerged in the last ten years, as evidenced by the numerous initiatives undertaken in this regard (Friday for Climate, Y20, among others) [48,49]. As a result of youth activism, the media and politicians have paid more attention to climate change issues, which has likely increased the level of concern among young people who rely on the internet and social media for their information. It is to be expected that today’s digital natives will rely less on printed sources and more on online resources than their older counterparts. However, the question of the validity of the sources consulted and the ability to understand such a vast and complex topic through the use of unstructured disclosures remains open [50,51,52,53]. In fact, the concept of sustainability was not considered easy to understand by most of the interviewees, compounded by the fact that it is an abstract and still open concept [6,25]. In particular, the interviewees perceived sustainability as constituted by the main pillar, the environmental dimension (representing 47% of the sustainability terms selected) and by two accessory pillars (10.7% social terms and 5.2% economic terms) that the young people interviewed regarded as playing a lesser role. This finding is particularly true when it comes to the idea of sustainability arising spontaneously in the minds of the respondents, as this concept was partially balanced in its three components when they attributed levels of importance to the benefits that sustainability brings to society (9.1 for environmental aspects, 8.4 for social aspects, 7.9 for economic aspects). The Argentine Zoomer consumers interviewed exhibited a moderately positive view of the sustainability of national production systems, with better ratings for vegetable production, intermediate ratings for extensive animal production, and lower ratings for intensive production. Also, in this case, the data reflect the information circulating in the media, which attributes high responsibility to livestock production for contributing to climate change [54], although from a scientific point of view there is no agreement on the actual greater sustainability of extensive livestock production compared to highly efficient intensive production [55], and there may be highly different impacts even within the same type of farming in different parts of the world [56]. In general, despite lower sustainability ratings than vegetable products, meat products get a relatively positive rating from Argentinian consumers, this could be due to the relatively low cost of meat and the country’s culinary tradition which sees a total consumption of animal protein in Argentina higher than the average of the Mercosur countries and one of the highest in the world [57]. In 2019, the apparent annual per-capita consumption was higher than 109 kg (14.6 kg of pork, 43.2 kg of chicken, and 51.6 kg of beef) [58].

The firm belief on the part of Zoomers that products from sustainable agriculture are healthier, safer and of higher quality, together with a greater ability to understand the concept of sustainability, plays a fundamental role in determining purchasing choices, as already highlighted in the literature [8], and Zoomers also show high ethical awareness, as shown by their food choices without animal proteins. Young consumers believe that market forces must support sustainable agriculture by acting in the same way as many of them already do, that is, making sustainable choices. However, they also endorse the development of certifications that identify products from sustainable agriculture, at prices similar to those from conventional agriculture. This point could open a wide debate on the actual cost of the products, and it could be argued that the cost of pollution should be borne by those responsible for it and that their products should be marketed at a higher price, and vice versa, the products that have a lower impact on natural resources should cost less. This fascinating topic, however, is not the subject of this work and, therefore, cannot be addressed here in its complexity.

However, as emerged from an extensive review [36], we can reiterate that budget constraint is one of the major deterrents in choosing more environmentally friendly products and, in this study, the phenomenon seems also to be confirmed for more sustainable products. Unlike what emerges from the literature regarding the social desirability bias in the respondents’ statements [59], we can highlight how in the present study the interest in more sustainable products is higher among those who have already made ethical purchasing choices. The psychological phenomenon of responding in a politically correct way is thus overcome.

## 5. Conclusions

Zoomers demonstrate a high degree of concern about the sustainability of the planet, yet their knowledge of sustainability is limited. Current and future young consumers making purchasing decisions can benefit from an effort to increase knowledge about “sustainable production”, which involves environmentally friendly, profitable, socially just and ethically sound production practices. Underlining that sustainability is based on three pillars and not only on the environmental one, could favour wider ethical choices.

Although young consumers express concern for the environment and an interest in applying sustainability principles to the food industry, they may need further explanation and guidance to fully grasp the notion. Concern for the planet alone does not seem to be sufficient to determine the choice to purchase more sustainable products. Young consumers, who are most aware of the benefits that sustainability brings to the quality of products and who better understand the concept, are also those who are more determined to buy them and who, for the most part, have made food choices in accordance with their ethical convictions. This suggests that improving knowledge is a key strategy to promoting food production that is respectful of the planet and favours the conscious choices of present and future buyers and purchase decision-makers. These results can be useful for stakeholders bringing their more sustainable products to market. Future research could analyse the characteristics of those who may have a greater willingness to buy more sustainable products in order to identify targeted marketing levers.

However, it is equally important to act on a public and international scale to define sustainability, give trustworthy information to citizens, reward successful businesses that are also environmentally conscious, and prevent further confusion about the topic. Given that the indication of sustainability is a good idea for the consumer, as it influences the selection process despite its current fuzziness, it is crucial to prevent the development of deceitful indications, claims and labels.

The study’s limitations consist of two elements: (1) focusing the study on a well-defined category of consumers, generation Z, does not allow comparisons to be made with the other generations and thus to identify distinctive behaviour of this cluster of consumers with respect to the others, (2) having analysed the consumers of only one country without having made a comparison with others makes the study partial in the interpretation of the results. These two aspects also represent future developments of the research; in fact, in order to investigate in detail the perceptions and attitudes of young consumers, it will be necessary to explore elements of diversity and communion with the older generations in order to identify on which distinctive elements to work, through the use of dedicated marketing strategies, on the side of the economic actors and on policy measures to favour sustainable transition, and on the side of the policy makers involved in the drafting of measures aimed at stimulating or better favouring sustainable food consumption.

## Figures and Tables

**Table 1:** Set of statements about the three dimensions of sustainability.

Sustainability Dimensions	Statements
Social dimension	1. Allows social development and roots in the local territory
	2. Respects the human rights of producers and workers
	3. Protects the public health of people
	4. Requires more training and work to reduce the human impact on the environment
Environmental dimension	5. Balances the development of humanity and care for the environment
	6. Maintains natural resources over time, for present and future generations
	7. Adopts low-polluting production processes (e.g., less use of chemicals)
	8. Favours biodiversity and reduces environmental risks (e.g., erosion, floods, fires, etc.)
Economic dimension	9. Is easier to implement on small production scales (e.g., family farming)
	10. Requires more labour than traditional agriculture
	11. Is a profitable activity that creates jobs
	12. Strives to reduce losses to make more efficient use of resources

**Table 2:** Demographic characteristics of respondents.

*n* = 537 Young People Aged between 18 and 27 “Zoomers”	
Gender	
Female	67.2%
Male	33.8%
Employment	
Students	17%
Students and workers	31%
Workers	52%
Education Level	
High school	45%
University students	26.7%
University degree	28.3%
Household	
1	21.8%
2	22.2%
3+	66%
Type of diet	
Omnivore	72.8%
Predominantly Carnivorous	10.6%
Flexitarian (*)	7.7%
Vegetarian	6.5%
Vegan	2.4%

Note: (*) Flexitarian is a flexible person in his/her food choices, predominantly vegetarian, but in social events such as at the home of friends, relatives and restaurants, agrees to eat dishes that contain animal proteins.

**Table 3:** Concern for the planet and resources.

Topics	Level of Concern (%)		
	Low	Moderate	High
Concern for the sustainability of the planet	1.1	11.0	87.9
Concern for the current food production system, since it does not preserve resources in the long term	1.7	9.5	88.8

**Table 4:** List of words related to sustainability, ordered by dimensions (*).

Aspect	Counts	%	Terms	Counts	%
Environmental	798	47.0	Environmental care	312	18.5
			Ecology	148	9.2
			Natural resources	298	19.4
Social	172	10.7	Social responsibility	62	3.8
			Social conscience	39	2.4
			Ethical work	71	4.4
Economic	83	5.2	Fair trade	29	1.8
			Economically feasible	36	1.0
			Corporate responsibility towards society	18	2.4
Crosscutting (**)	598	37.1	Enduring over time	331	20.5
			Recycle	155	9.6
			Commitment to future generations	112	7.0
Total answers	1611	100.0		1611	100.0

Note: (*) The list of words was chosen based on a previous study by the authors [44]. (**) Crosscutting words may refer to two or three aspects.

**Table 5:** Level of agreement with the statement “The concept of sustainability is clear and easy to understand for young people”.

Question	Level of Agreement in %		
The concept “sustainability” is clear and easy to understand for young people	Low 22.0	Moderate 45.2	High 32.8

**Table 6:** Proposed statements about the three dimensions of sustainability.

Statements on Sustainability	Average	Sustainability Dimension	Average Valuation of the Dimension
1. Allows social development and roots in the local territory	7.9	Social dimension	8.4
2. Respects the human rights of producers and workers	7.9		
3. Protects the public health of people	8.6		
4. Requires more training and work to reduce the human impact on the environment	9.0		
5. Balances the development of humanity and care for the environment	9.2	Environmental dimension	9.1
6. Maintains natural resources over time, for present and future generations	9.3		
7. Adopts low-polluting production processes (e.g., less use of chemicals)	8.8		
8. Favours biodiversity and reduces environmental risks (e.g., erosion, floods, fires, etc.)	9.1		
9. Is easier to implement on small production scales (e.g., family farming)	7.9	Economic dimension	7.9
10. Requires more labour than traditional agriculture	7.2		
11. Is a profitable activity that creates jobs	8.4		
12. Strives to reduce losses to make more efficient use of resources	8.3		

**Table 7:** Level of agreement with the factors that could favour sustainable food production (%).

Statement	Level of Agreement (%)		
	Low	Moderate	High
Consumers, through their purchase choices, drive a change in the current way of producing towards more sustainable production of food.	11.7	29.1	59.2
Consumers agree to pay a premium price for sustainable food.	25.3	28.7	46.0
Certification should be issued to help consumers recognise sustainable food.	16.0	29.1	54.2
Sustainable production of food is promoted by the State (e.g., policies, laws).	28.3	23.5	48.2

**Table 8:** Comparison between the perceptions of the “Undecided” and the “Determined” consumers concerning the characteristics of sustainable products.

Statement	Would You Be Willing to Buy Sustainable Products?		
	Undecided	Determined	Sig.
Sustainable food is tastier than conventional food currently on the market.	5.56	7.25	<0.001
Sustainable food is “healthier”.	7.37	8.18	0.006
Sustainable food is “safer” because it undergoes more control.	6.62	7.49	0.01
Sustainable food is of “higher quality” than traditional products on the market.	6.58	8.08	<0.001
Sustainable food is “more expensive” than conventional food on the market.	7.92	7.80	0.661

## Data Availability

Data is contained within the article or Supplementary Materials.

## Supplementary Materials

The following supporting information can be downloaded at: https://www.mdpi.com/article/10.3390/foods12051019/s1, Table S1: ANOVA test; Table S2: Robustness tests of Welch and Brown-Forsythe; Table S3: ANOVA test.
