# Supplementary material for: Perceptions and Attitudes of Argentine Zoomers towards Sustainable Food Production
Source: Foods. 2023 Feb 27;12(5):1019. doi: 10.3390/foods12051019 (PMC10000960; doi:10.3390/foods12051019)
Supplement: Supplementary file 1 [file foods-12-01019-s001.zip › foods-2220936-supplementary.pdf]

foods

MDPI

Supplementary Materials

Table S1. ANOVA test.

ANOVA						
		**Sum of Squares**	**df**	**Mean Square**	**F**	**Sig.**
Social dimension	Between Groups	0.114	1	0.114	4.728	0.031*
	Within Groups	6.220	258	0.024		
	Total	6.334	259			
Environmental dimension	Between Groups	0.692	1	0.692	32.771	0.000*
	Within Groups	5.449	258	0.021		
	Total	6.141	259			
Economic dimension	Between Groups	0.220	1	0.220	9.152	0.003*
	Within Groups	6.200	258	0.024		
	Total	6.420	259			

* Significance $p < 0.05$.

Table S2. Robustness tests of Welch and Brown-Forsythe.

Robustness Tests of Equality of Means					
		**Statistic**	**df1**	**df2**	**Sig.**
Social dimension	Welch	4.607	1	233.496	0.033
	Brown-Forsythe	4.607	1	233.496	0.033
Environmental dimension	Welch	35.621	1	208.895	0.000
	Brown-Forsythe	35.621	1	208.895	0.000
Economic dimension	Welch	9.382	1	257.563	0.002
	Brown-Forsythe	9.382	1	257.563	0.002

Table S3. ANOVA test.

ANOVA<br>The concept of sustainability is "easy to understand" for everyone					
	Sum of Squares	df	Mean Square	F	Sig.
Between Groups	44.482	1	44.482	5.760	0.017*
Within Groups	1992.329	258	7.722		
Total	2036.812	259			

* Significance $p < 0.05$.
